# Supplementary material for: The Effect of Phonomyography Prototype for Intraoperative Neuromuscular Monitoring: A Preliminary Study
Source: Bioengineering (Basel). 2024 May 14;11(5):486. doi: 10.3390/bioengineering11050486 (PMC11117765; doi:10.3390/bioengineering11050486)
Supplement: Supplementary file 1 [file bioengineering-11-00486-s001.zip › bioengineering-2977041-supplementary.pdf]

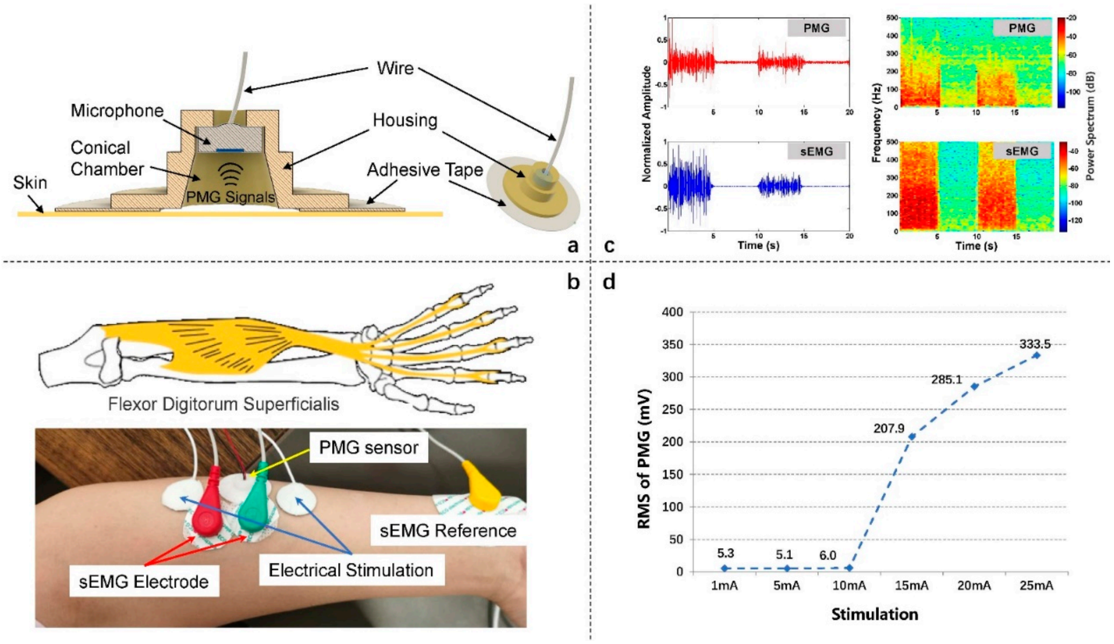

Figure S1. Schematic representation and validation of the PMG prototype. (a)The illustration of PMG sensing with a conical acoustic chamber in the housing to eliminate ambient noise [1,2]. (b)The experimental setup to monitor neuromuscular activities using synchronous PMG and surface electromyography (sEMG), and the muscle (Flexor Digitorum Superficialis, FDS) contraction can be selectively induced by electrical stimulation. (c)Time-domain waveform and time-frequency information of PMG and sEMG signals during voluntary muscle contraction [3]. (d)The relation between PMG Root Mean Square (RMS) and the intensity of electrical stimulation without voluntary muscle contraction, the data is from the FDS muscle of a typical subject. It is important to note that sEMG is contaminated by the artifacts of electrical stimulation, thus sEMG is not suitable for the measurement of depth of muscle relaxation throughout the peri-operative period. The phenomena of subgraph (d) indicate that PMG is immune to electrical stimulation artifacts since no PMG response is observed under the influence of low level (i.e., below 10 mA) electrical stimulation, and once the low-frequency vibration of myofiber is evoked by electrical stimulation, the intensity of PMG is positively increased with stimulation.

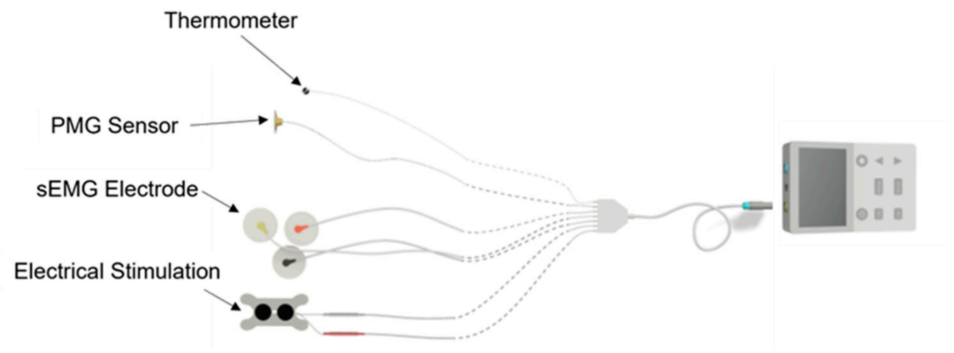

Figure S2. Schematic of the PMG prototype.

References

1. Guo W.C., Fang Y., Sheng X.J. and Zhu X.Y., "Measuring motor unit discharge, myofiber vibration and haemodynamics for enhanced myoelectric gesture recognition." IEEE Transactions on Instrumentation and Measurement, 2023, 72: 4001510.

2. Guo W.C., Sheng X.J. and Zhu X.Y., "Assessment of muscle fatigue based on motor unit firing, muscular vibration and oxygenation via hybrid mini-grid sEMG, MMG, and NIRS sensing." IEEE Transactions on Instrumentation and Measurement, 2022, 71: 4008010.

3. Guo W.C., Sheng X.J., Liu H.H. and Zhu X.Y., “Mechanomyography Assisted Myoeletric Sensing for Upper-extremity Prostheses: a Hybrid Approach”, IEEE Sensors Journal, 2017, 17(10):3100-3108.
